# Case Report: Persistent fifth aortic arch with coarctation and fourth aortic arch interruption causing infant heart failure – the prenatal and postnatal echocardiographic course

**DOI:** 10.3389/fmed.2026.1802083

**Published:** 2026-05-22

**Authors:** Xiaohong Zhang, Guozhen Yuan, Kunpeng Li, Yangcan Duan, Junli Hu, Dongchen Fan

**Affiliations:** 1 Department of Ultrasound, Affiliated Hospital of Jining Medical University, Jining, China; 2 Jining Medical University, Jining, China

**Keywords:** aortic coarctation, heart failure, interruption of aortic arch, persistent fifth aortic arch, prenatal ultrasound

## Abstract

Persistent fifth aortic arch (PFAA) with concomitant interruption of the fourth aortic arch is an extremely rare congenital aortic arch malformation. Due to its unique anatomical configuration, variable hemodynamic presentations, and the potential for adequate fetal circulatory compensation in some cases, prenatal ultrasonographic diagnosis remains challenging. This condition is frequently misidentified as a normal variant or confused with other aortic arch anomalies. We report a male infant with unremarkable prenatal ultrasound who presented at 90 days of life with a 12-days history of worsening cough. Postnatal echocardiography confirmed the diagnosis of PFAA with coarctation and Type A interruption of the fourth aortic arch. Resection of the coarctation segment and reconstruction of the aortic arch using an autologous pericardial patch were performed, along with end-to-side anastomosis between the fourth arch and the descending aorta. One year postoperatively, echocardiography revealed unobstructed aortic arch flow without pressure gradient and recovery of left ventricular function. Through a systematic analysis of prenatal sonographic features, postnatal clinical progression, imaging evolution, and surgical outcomes, this case provides insights into prenatal recognition, postnatal management, and clinical decision-making for this rare aortic arch malformation.

## Introduction

Persistent fifth aortic arch (PFAA) is a rare congenital vascular anomaly, anatomically characterized by an extrapericardial abnormal vessel. This vessel typically originates from the ascending aorta, situated opposite or adjacent to the orifice of the brachiocephalic artery, and ultimately connects to either the descending aorta or the pulmonary artery system (1). From an embryological perspective, six pairs of branchial arch arteries sequentially form between the ventral and dorsal aortae, subsequently undergoing remodeling and regression. The fifth aortic arch pair typically regresses completely or may not develop in some embryos; failure of regression leads to persistence of the fifth aortic arch (2).

Persistent fifth aortic arch coexisting with interruption of the fourth aortic arch represents a subtype of PFAA. In this anomaly, the brachiocephalic artery, left common carotid artery, and left subclavian artery arise from the fourth arch, the distal end of which terminates in a blind pouch. The PFAA connects the ascending aorta to the descending aorta (establishing a systemic-to-systemic connection). The natural history and hemodynamic impact of this combined anomaly are not yet fully understood. During the fetal period, the PFAA may exhibit no significant abnormalities in lumen diameter or hemodynamics, with coarctation signs remaining subtle, potentially leading to missed diagnoses (3). However, postnatal closure of the ductus arteriosus and subsequent changes in blood flow patterns can precipitate rapid narrowing of the PFAA shortly after birth, potentially triggering progressive heart failure and life-threatening complications.

Current literature primarily consists of case reports or small case series, with most diagnoses occurring in the neonatal or infant period (4, 5). Prenatal ultrasound diagnosis of this condition is extremely rare, with only three English-language case reports identified to date (3, 6, 7), reflecting a very low prenatal detection rate. This indicates a very low prenatal detection rate, likely due to the absence of significant hemodynamic changes during fetal life, combined with limited awareness among sonographers of this specific aortic arch anomaly. Therefore, a deeper understanding of the prenatal sonographic features and postnatal clinical course of this combined anomaly is crucial for achieving early diagnosis, timely intervention, and improved prognosis.

This report describes a case of severe heart failure in an infant caused by coarctation of a PFAA combined with interruption of the fourth aortic arch. Through a comprehensive analysis of its prenatal ultrasound presentation, postnatal clinical progression, imaging evolution, and outcomes following surgical intervention, this case aims to provide valuable insights for the diagnosis and management of this rare aortic arch anomaly.

## Case presentation

A 90-days-old male infant, born at 40 weeks via vaginal delivery, had an unremarkable neonatal course. The infant was admitted to the hospital due to persistent coughing for 12 days, with worsening symptoms over the last 2 days. The mother was 28 years old and a first-time gravida, with no history of gestational diabetes, hypertension, or autoimmune-related diseases. Non-invasive prenatal DNA testing during pregnancy was normal, and routine prenatal ultrasound scans showed no abnormalities.

### Physical examination findings upon admission

The patient weighed 6.5 kg, with a body temperature of 36.0 °C. The upper limb blood pressure was 78/45 mmHg (1 mmHg = 0.133 kPa). The heart rate was 148 beats/min, and the respiratory rate was 42 breaths/min. The percutaneous oxygen saturation (SpO2) was 99.3%. The patient was conscious with fair responsiveness. Mild pharyngeal congestion was observed, without visible herpes. Breath sounds in both lungs were coarse, with no dry or moist rales. A grade II systolic murmur was audible in the precordial region. The abdomen was soft, with the liver and spleen not palpable below the costal margin. The capillary refill time of the fingers was <2 s.

### Laboratory findings

CK-MB was 5.25 ng/ml, LDH 287.3 U/L, α-HBDH 213.1 U/L, BNP 1681 ng/L, high-sensitivity troponin 77 ng/L, and hemoglobin 84 g/L. The remainder of the routine blood tests, C-reactive protein, liver and kidney function, and coagulation function were unremarkable.

### Electrocardiogram

The 12-lead electrocardiogram (ECG) (paper speed 25 mm/s, gain 10 mm/mV) showed sinus tachycardia (heart rate 188 beats/min), a QTc interval of 506 ms, left ventricular high voltage (RV_5_ + SV_1_ = 5.84 mV), accompanied by increased P-wave amplitude, an abnormal ptfV_1_ (ptfV_1_ < −0.04 mm⋅s), and T-wave changes in multiple leads (Supplementary Figure 1).

### Imaging findings upon admission

**FIGURE 1:**
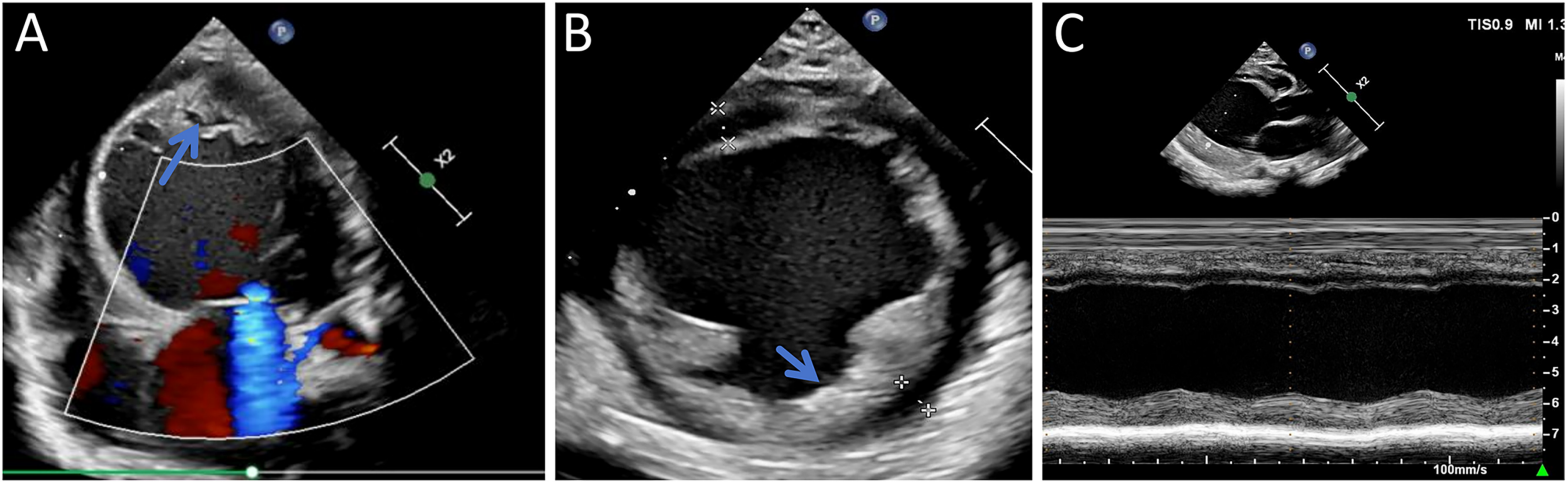
Preoperative imaging. **(A)** Four-chamber view showing spherical left ventricular enlargement with increased apical trabeculations (blue arrow). **(B)** Short-axis view demonstrating thickening of the interventricular septum and left ventricular free wall with increased endocardial echogenicity (blue arrow). **(C)** Global left ventricular wall motion discoordination with diffusely reduced systolic contraction.

**FIGURE 2:**
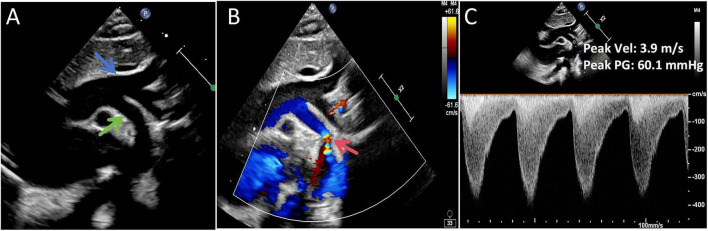
Preoperative imaging. **(A)** Long-axis view of the aortic arch showing the fourth aortic arch (blue arrow) giving rise to three supra-aortic branches and terminating blindly. The persistent fifth aortic arch (green arrow) is seen connecting to the descending aorta. **(B)** Localized stenosis of the PFAA with a color mosaic flow pattern at the stenotic segment (red arrow). **(C)** Peak systolic flow velocity at the stenotic site measuring 3.9 m/s.

Chest X-ray revealed an enlarged cardiac silhouette (Supplementary Figure 2), while lung ultrasoundgraphy showed irregular pleural lines bilaterally, with lung sliding sign present. A-lines were observed in both lung fields, and a few comet tail signs were noted in the I and II lung zones. In the right lung, a 2–3 rib fusion B-line was observed in zone V. No pleural effusion was noted bilaterally. Echocardiography results were as follows: the left ventricular end-diastolic anterior-posterior diameter was 39 mm, showing spherical dilation (Figure 1A). Left ventricular wall thickening was noted, with a septal thickness of 6 mm and posterior wall thickness of 6 mm. Left ventricular endocardial and trabecular thickening with enhanced echogenicity was observed, especially in the posterior wall (Figure 1B). There was asynchronous ventricular wall motion with overall reduced systolic function (Figure 1C), and the LVEF measured by the biplane Simpson’s method was 25%. Pulsed-wave Doppler of the mitral valve orifice showed an E-wave velocity of 1.4 m/s, an A-wave velocity of 0.4 m/s, an E/A ratio > 2, a deceleration time (DT) of the E-wave of 65 ms, and a markedly steep descending limb of the E-wave. Tissue Doppler imaging (TDI) showed an e’ velocity of 0.05 m/s at the septal mitral annulus. These findings indicated severely impaired left ventricular diastolic function. Further investigation revealed a left-sided aortic arch anomaly: a persistent fifth aortic arch with severe narrowing, and a discontinuation of the fourth arch after giving off the brachiocephalic trunk, left common carotid artery, and left subclavian artery. The fifth arch was connected to the descending aorta, with a stenotic region approximately 2 mm in diameter, with color mosaic flow (Figures 2A, B). Forward blood flow velocity was significantly increased, with a maximum velocity (Vmax) of 3.9 m/s (Figure 2C). The abdominal aortic spectrum showed small slow-wave changes, and mild to moderate mitral regurgitation was detected. The foramen ovale was patent, but no other obvious anomalies were seen in the heart or coronary artery openings. Ultrasound findings confirmed the presence of an aortic arch anomaly, characterized by a narrowed PFAA with Type A interruption of the fourth aortic arch. Additional findings included left ventricular dilation, impaired left ventricular function, mild-to-moderate mitral regurgitation, and a patent foramen ovale.

### Follow-up and surgical intervention

**FIGURE 3:**
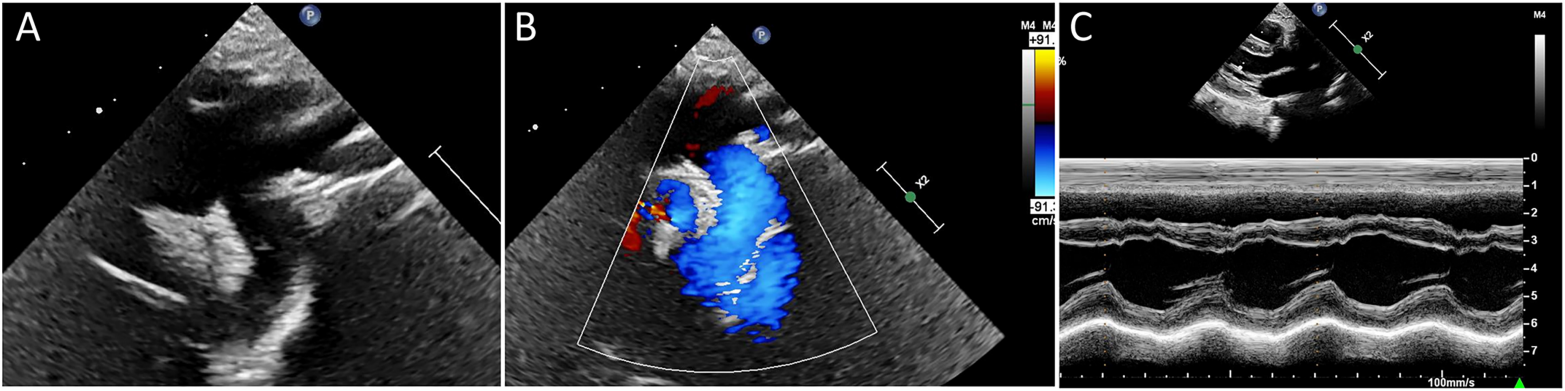
One-year postoperative follow-up imaging: **(A)** Postoperative imaging after fourth arch and descending aorta reconstruction showing normal vessel diameter. **(B)** Patency of blood flow in the descending aortic arch. **(C)** Normalized wall thickness and coordinated systolic motion of the ventricular walls.

At 95 days of age, the infant was transferred to the Fuwai Hospital of the Chinese Academy of Medical Sciences for surgical treatment. Intraoperatively, an abnormal aortic arch development was observed, with a persistent fifth aortic arch accompanied by severe coarctation. The fourth arch gave rise to the brachiocephalic trunk, left common carotid artery, and left subclavian artery, and then terminated. The PFAA along with the adjacent narrowed portion was resected. After longitudinal incision of the proximal descending aorta, the aortic arch was widened and reconstructed using an autologous pericardial patch. Subsequently, an incision matching the diameter of the descending aorta was created in the fourth aortic arch, followed by an end-to-side anastomosis between the fourth aortic arch and the descending aorta. Postoperatively, unobstructed blood flow was established in the aortic arch and descending aorta, with gradual improvement of LVEF. On postoperative day 9, LVEF increased to 63%, and the infant was discharged. At the 1-year follow-up, echocardiography demonstrated a left ventricular end-diastolic anterior–posterior diameter of 26 mm, interventricular septal thickness of 4 mm, and left ventricular posterior wall thickness of 4 mm, with coordinated segmental wall motion and normal systolic contraction. LVEF further improved to 68%, and the descending aortic arch remained patent, with a peak velocity of 1.3 m/s (Figure 3).

### Review of prenatal ultrasound

**FIGURE 4:**
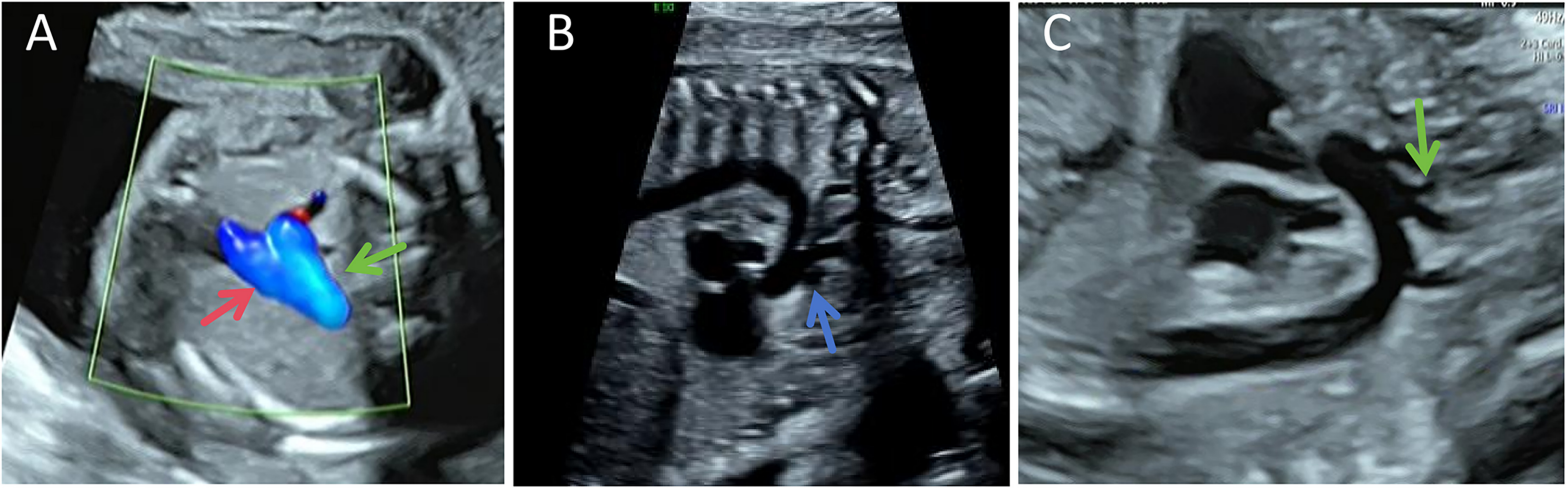
Prenatal imaging review. **(A)** Fetal three-vessel view demonstrating normal diameters and flow directions of the ductus arteriosus (red arrow) and aortic arch (green arrow). **(B)** Aortic arch long-axis view showing the brachiocephalic trunk and branches originating from the ascending aorta (blue arrow). The diameter of the PFAA is unremarkable. **(C)** Aortic arch long-axis view in a fetus with bovine arch variant, illustrating a common trunk origin of the brachiocephalic trunk and left common carotid artery.

Routine prenatal ultrasound examinations performed at 24 weeks and 3 days and at 30 weeks of gestation demonstrated normal alignment, diameter, number, and flow direction in the three-vessel view (Figure 4A). The three-vessel view showed normal aortic arch diameter and flow velocity. In the long-axis view of the aortic arch, the brachiocephalic trunk, left common carotid artery, and left subclavian artery were observed to originate from the fourth aortic arch, the distal portion of which terminated in a blind end. The PFAA connected to the descending aorta, and no significant abnormalities in diameter or flow velocity were detected in the descending aorta (Figure 4B). The four-chamber view, left ventricular outflow tract view, and right ventricular outflow tract view were all unremarkable. Because the bovine aortic arch variant–characterized by a common origin of the brachiocephalic trunk and left common carotid artery–is relatively common in clinical practice (Figure 4C) and is typically asymptomatic after birth, the aortic arch anatomy was misinterpreted as a benign anatomical variant of a common trunk. Consequently, insufficient attention was paid to this finding, no further fetal echocardiographic evaluation was performed, and a definitive prenatal diagnosis was not established.

## Discussion

The exact incidence of PFAA remains uncertain. According to a single-center study, the estimated incidence was 0.01% among children who underwent echocardiography due to factors such as heart murmurs or cyanosis (4); however, the true incidence in the general pediatric population is likely lower than this value. The occurrence of PFAA combined with fourth aortic arch interruption is even rarer. This report presents a rare case of late-onset severe heart failure in infancy due to PFAA combined with Type A fourth aortic arch interruption. The clinical peculiarity of this case lies in the delayed onset of heart failure, which manifested at 90 days of age following an asymptomatic neonatal period. This delayed presentation may be attributed to the PFAA temporarily maintaining systemic perfusion as a compensatory pathway. However, progressive stenosis of the PFAA ultimately resulted in hemodynamic decompensation. Notably, routine prenatal ultrasound failed to establish a definitive diagnosis, and the aortic arch morphology was misinterpreted as a bovine-type aortic arch. This underscores the diagnostic challenge of such malformations during fetal life, when hemodynamic abnormalities may be subtle or absent, leading to missed diagnoses.

The bovine aortic arch is a common anatomical variant of the aortic arch, characterized by the origin of the brachiocephalic trunk and left common carotid artery from a common trunk at the aortic arch (8). This variant is often observed during routine prenatal ultrasound. Studies suggest that this variant may be associated with the development of thoracic aortic aneurysms in adulthood (9). However, in the adolescent phase, hemodynamics typically show no significant abnormalities, and there is currently no clear evidence linking this variant to specific genotypes. Therefore, in clinical practice, a simple bovine aortic arch detected prenatally is often regarded as a benign variation with a good prognosis (10). In prenatal ultrasound differentiation, the three-vessel view is of limited value, because both PFAA with type A interruption of the fourth aortic arch and bovine aortic arch may appear normal on this view. The key differentiation lies in the long-axis view of the aortic arch: the bovine aortic arch is characterized by a “two-vessel common trunk” (brachiocephalic trunk and left common carotid artery originating from a common trunk), while PFAA with fourth aortic arch interruption presents with a “three-vessel common trunk” (brachiocephalic trunk, left common carotid artery, and left subclavian artery all originating from the fourth arch). Thus, accurate identification of the vessel configuration in the long-axis view of the aortic arch is crucial for differentiating these two conditions.

Moreover, this case suggests that although the diameter and hemodynamic indices of PFAA may fall within the normal range in prenatal examinations, there remains a risk of progressive stenosis after birth. One study, through histological analysis of a PFAA autopsy specimen, found that its vascular wall structure resembled that of the ductus arteriosus (11). This finding may explain why some PFAA infants develop aortic stenosis shortly after birth, despite normal prenatal ultrasound findings: similar to the ductus arteriosus, PFAA may undergo construction and remodeling after birth due to environmental factors such as changes in hemodynamics and blood oxygen levels, leading to rapid progression of stenosis in the immediate postnatal period. Therefore, postnatal echocardiographic follow-up is clinically essential for observing intracardiac structures, dynamically monitoring cardiac function, and assessing hemodynamic changes at the site of coarctation. However, conventional echocardiography has limitations in identifying complex extracardiac anatomical variations, posing a risk of misdiagnosis (5). Computed tomography angiography offers significant advantages in delineating vascular origin, branch course, and spatial relationships, thereby complementing echocardiography. The core value of cardiac catheterization lies in precisely measuring the trans-stenotic pressure gradient, confirming the blood flow pathway, and providing interventional opportunities (e.g., balloon dilation or stenting) in selected cases. Although traditionally regarded as the diagnostic gold standard, its invasive nature has led to its gradual replacement by noninvasive imaging techniques for purely diagnostic purposes (12).

Despite the critical preoperative state, the infant in this case achieved excellent outcomes following timely surgical intervention. Specifically, resection of the narrowed persistent fifth arch and end-to-side anastomosis between the fourth arch and the descending aorta successfully reconstructed a single patent aortic arch. Postoperatively, the left ventricular dimensions quickly returned to normal, and the ejection fraction fully restored to normal levels, effectively demonstrating the remarkable remodeling capacity of infant myocardium after acute pressure load relief. This outcome aligns with the prognosis observed in small case series reported in the literature (4), reinforcing a key clinical message: even with a delayed diagnosis resulting in severe heart failure, timely anatomical correction can lead to complete recovery of heart function and a good prognosis.

## Limitations

A limitation of this report is the lack of lower limb blood pressure measurements at initial evaluation due to its retrospective nature, which meant that the upper-lower limb blood pressure gradient could not be assessed preoperatively.

## Conclusion

During prenatal ultrasound examination, if a three-vessel common trunk originating from the ascending aorta is observed in the long-axis view of the aortic arch, consideration should be given to the possibility of a PFAA combined with Type A fourth aortic arch interruption. Although the diameter of the PFAA and hemodynamic abnormalities may not show significant narrowing or disturbances in prenatal imaging, there remains a risk of rapid stenosis in the short term after birth, which can lead to heart failure. Accurate identification on prenatal ultrasound, close echocardiographic follow-up after birth, and timely surgical intervention are crucial for improving the prognosis of affected infants.

## Data Availability

The original contributions presented in this study are included in the article/Supplementary material, further inquiries can be directed to the corresponding author.

## Supplementary material

Supplementary Figure 1 Sinus tachycardia, P-wave changes, T-wave changes in multiple leads.

Supplementary Figure 2 Chest radiograph showing cardiomegaly.
