# Supplementary figures and images for: Case Report: Persistent fifth aortic arch with coarctation and fourth aortic arch interruption causing infant heart failure – the prenatal and postnatal echocardiographic course
Source: Front Med (Lausanne). 2026 May 22;13:1802083. doi: 10.3389/fmed.2026.1802083 (PMC13237689; doi:10.3389/fmed.2026.1802083)

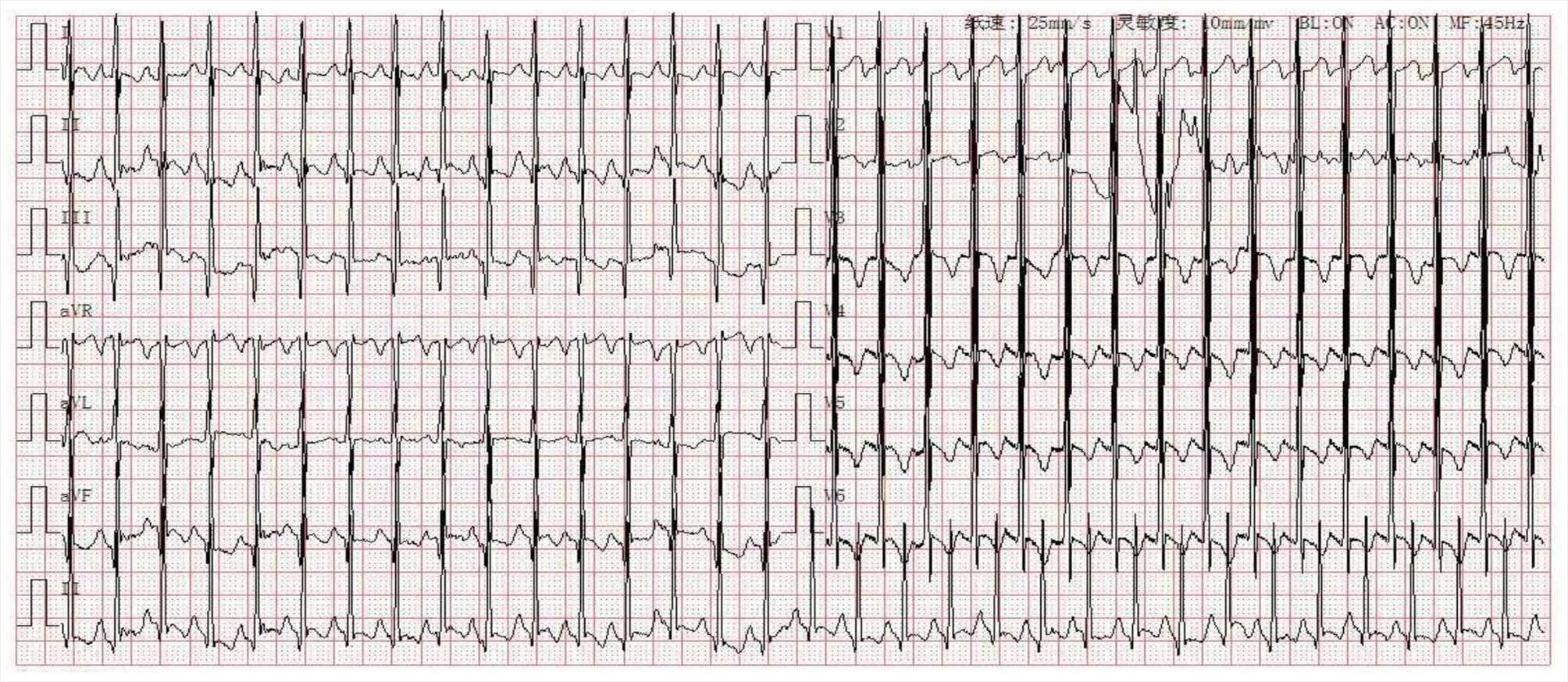

Supplement: Supplementary Figure 1 — Sinus tachycardia, P-wave changes, T-wave changes in multiple leads. [file Image_1.tif]

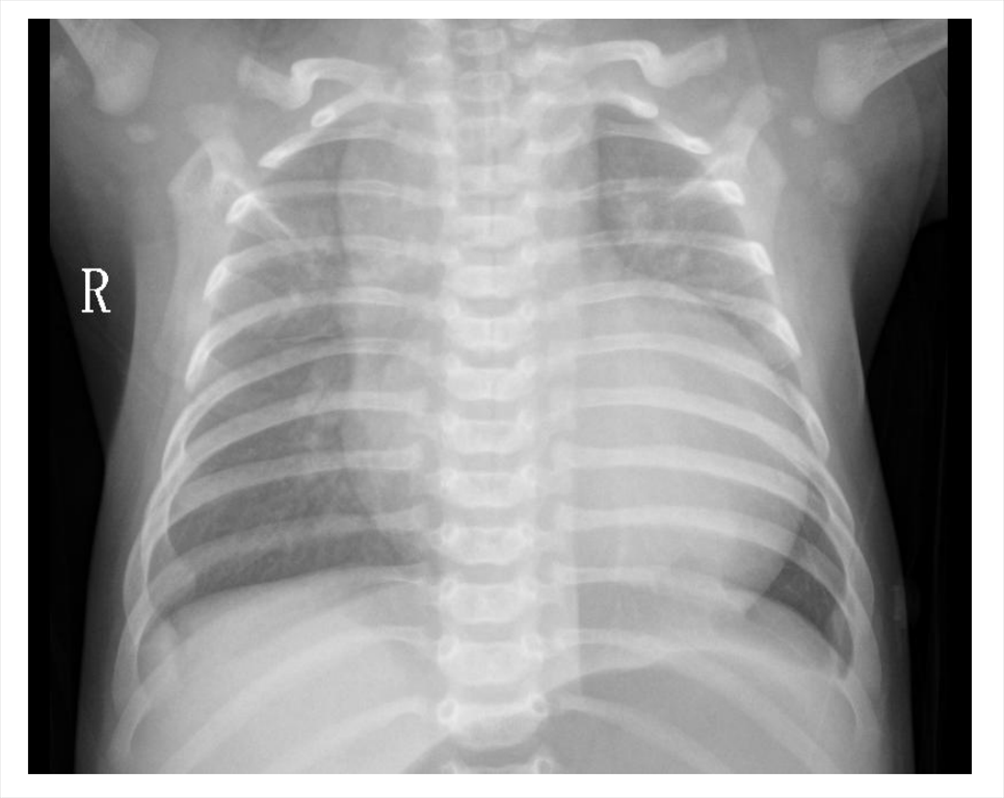

Supplement: Supplementary Figure 2 — Chest radiograph showing cardiomegaly. [file Image_2.tif]
